# Nutritional Status and Risk Factors for Frailty in Community-Dwelling Older People: A Cross-Sectional Study

**DOI:** 10.3390/nu12041041

**Published:** 2020-04-10

**Authors:** Pilar Pérez-Ros, Rafael Vila-Candel, Lourdes López-Hernández, Francisco Miguel Martínez-Arnau

**Affiliations:** 1 Department of Nursing, Universidad Católica de Valencia San Vicente Mártir, 46007 Valencia, Spain; rafael.vila@ucv.es (R.V.-C.); lourdes.lopez@ucv.es (L.L.-H.); 2 Department of Obstetrics and Gynaecology, Hospital Universitario de la Ribera, FISABIO. Crtra. Corbera km 1, 46600 Valencia, Spain; 3 Department of Nursing, Faculty of Nursing and Podiatry, Universitat de València. Jaume Roig, s/n, 46010 Valencia, Spain; 4 Department of Physiotherapy, Universitat de València. 46010 Valencia, Spain; francisco.m.martinez@uv.es; 5 Frailty and Cognitive Impairment Research Group (FROG), University of Valencia, 46010 Valencia, Spain

**Keywords:** frailty, nutritional status, independent living, risk factors, lifestyle, ageing, health

## Abstract

Objective: This study aims to assess the relationship that frailty has with nutritional status and functional risk factors in community-dwelling older adults. Methods: Cross-sectional study in community-dwelling older people, independent for walking and without impaired cognition. Frailty was assessed by Fried criteria. Nutritional status was analyzed by the Mini Nutritional Assessment Short Form (MNA-SF), biochemical markers (albumin, total proteins, cholesterol, lymphocytes, and hemoglobin); and anthropometric parameters (body mass index [BMI], body fat percentage, handgrip, and perimeters). A comprehensive geriatric assessment analyzed other risk factors: functionality, cognition, falls, comorbidity, polypharmacy, physical activity, and quality of life (QoL). Results: We included 564 elderly people with a mean age of 76.05 (standard deviation 3.97) years; 63.1% (*n* = 356) were women, and 83.9% (*n* = 473) were prefrail, and frail. The sample presented high functionality and a nutritional status with a predominance of overweight and obesity. Factors associated with frailty (*R*^2^ = 0.43) were age over 75 years (odds ratio [OR] 3.31, 95% confidence interval [CI] 1.76, 6.21; *p* < 0.001), female gender (OR 2.37, 95% CI 1.24, 4.52; *p* = 0.009), anemia (OR 2.45, 95% CI 1.19, 5.02; *p* = 0.015), falls (OR 1.94, 95% CI 1.12, 3.25; *p* = 0.016) and the fear of falling (OR 4.01: 95% CI 1.76, 9.16; *p* = 0.001). Performing more than 3 weekly hours of physical activity was found to be a protective factor (OR 0.23, 95% CI 0.15, 0.35; *p* < 0.001). Conclusions: The relationship between frailty and malnutrition in functionally independent community-dwelling older people is unclear. More studies are needed to know what nutritional markers are related to frailty, cognition, and functionality in order to discriminate the risk factors for community-dwelling older people at risk of malnutrition and dependency.

## 1. Introduction

Life expectancy is increasing in most countries globally. Currently, 9% of the world’s population is over 65 years old, and this figure is expected to increase to 16% by 2050 [1]. Population ageing is accompanied by increased comorbidity, functional decline, and increased prevalence of geriatric syndromes—complex health conditions that are not framed in specific morbidity pathologies but rather manifest through phenomena like frailty, falls, immobility, incontinence, and delirium [2].

Frailty is a syndrome characterized by diminished physiological reserves, and its prevalence is aggravated by age [3]. While there are several definitions of the syndrome, Rockwood et al. favor a multidimensional concept that encompasses the physical, psychological and social component and its comorbidity [4]. In contrast, the model proposed by Fried [5]—the most widely used worldwide —defines frailty as the presence of at least three of five physical indicators: weakness (reduced grip strength), slowness (reduced gait speed), weight loss, low physical activity, and exhaustion. People with one or two indicators are classified as prefrail. Prevalence of frailty at a community level is estimated at 3.9% to 51.4%, and prevalence of prefrailty ranges from 13.4% to 71.6%, depending on the geographical region and the screening tools used [6].

In clinical practice, people over 80 years of age with high comorbidity or disability have often been misidentified as frail [7]. Although they can overlap, comorbidity, loss of functionality and frailty are independent entities. At the same time, they all have negative effects on health and are associated with disability, hospitalization and death [8,9]. Functionality is defined as a set of physical and mental skills necessary for the maintenance of independence in the performance of basic and instrumental activities of daily living as well as in social and cognitive aspects [10]. Prefrailty and frailty are risk factors for loss of function, although this relationship is closer in people aged over 75 years [11,12].

**Figure 1:**
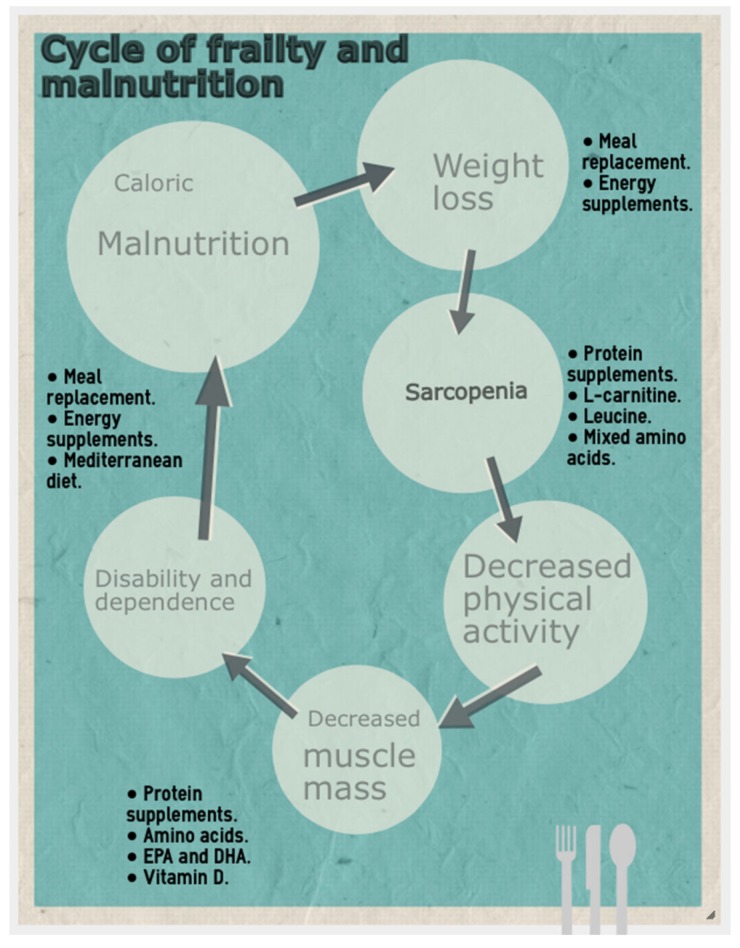
Frailty development cycle, adapted from Fried et al. [5].

The phenotype proposed by Fried [5] (Figure 1) frames sarcopenia secondary to malnutrition as contributing to low physical activity and increased dependence. Both entities, obesity and undernutrition, are related to sarcopenia and frailty although these conditions are more prevalent in residential and hospital settings than in the community [13,14]. Both the quantitative assessment (energy intake) and the qualitative assessment (nutrient quality) are important because the lack of micronutrients (Vitamin D or leucine) and macronutrients (proteins) are considered risk factors for frailty, while the type of diet (Mediterranean diet) can prevent or reverse frailty [15]. In addition, nutritional screening using the Mini Nutritional Assessment (MNA) or Short Form MNA (MNA-SF) is recommended for the detection of malnutrition [16,17]. On the other hand, some studies report that undernutrition is not the only nutritional state related to frailty; some parameters for overnutrition, such as elevated BMI, high body fat percentage and central obesity in adulthood could also increase risk in aging [18,19].

International guidelines agree on the need to carry out a correct nutritional assessment, although the subsequent intervention strategies are not really defined due to difficulties in implementation [20,21,22]. The lack of global consensus on ranges and parameters of normality, the diversity of assessment tools in each parameter of nutritional assessment [23], and the diversity in the assessment of frailty [24] all pose challenges for prevention strategies. In addition, a complete nutritional assessment includes dietary, clinical and anthropometric assessments; screening tools; and analyses of biochemical markers, requiring a considerable investment of time and resources, as well as interdisciplinary work [25]. Comparisons between some nutritional parameters are problematic, as body fat percentage increases with age, aennd body distribution differs by sex and could modify the waist circumference. Loss of height with age could also modify BMI. Taken together, these aspects complicate reaching a consensus on what is “normal” and identifying clear risk factors linked to frailty. The increase in body fat and loss of lean mass is related to the ageing process [19]. So, there is a dearth of longitudinal studies that could elucidate the role of nutrition in reversing frailty or that analyze the nutritional status of other populations [14,16].

A recent systematic review [20] reported a prevalence of frailty of 13.6% and of prefrailty, 30.9% in previously robust elderly, concluding that community-dwelling older adults are prone to developing frailty. Increased awareness of the factors that confer high risk of frailty in this population subgroup is vital for informing the design of interventions to prevent frailty and minimize its consequences.

The aim of the study was to determine the factors associated with frailty in community-dwelling older people with functional independence and no cognitive impairment, and to analyze their relationship with nutritional status.

## 2. Materials and Methods

### 2.1. Study Design and Participants

A cross-sectional study was carried out from 1 December 2014 to 31 May 2015. The inclusion criteria were as follows: participants aged 70 years or older; independent for walking (with possible technical aids, but not assisted by another person); and living within the catchment area of La Ribera Health Department (Valencia, Spain). Exclusion criteria were: refusal to participate in the study, the existence of associated disease conditions resulting in a life expectancy of under 6 months, blindness and deafness, serious psychiatric problems (severe depression subjected to treatment or acute psychosis), or moderate to severe cognitive impairment (diagnosed previously by a physician) and/or after cognitive evaluation with the Mini Mental State Examination (MMSE) with Cronbach’s alpha 0.90 and a score of 24 points or less, indicating cognitive impairment [26]. Loss of function is increased in both frail and prefrail older adults [12], so we dichotomized participants into a robust group and a prefrail/frail group, according to Fried’s phenotype of frailty [5]. Recruitment covered the period from December 2014 to May 2015. It was carried out in the health centers and social centers for older adults in the region of La Ribera. 

### 2.2. Sample Size Description

The sample size was calculated based on the population census of 2013. The region of La Ribera (Valencia, Spain) has a population of about 253,330 inhabitants (15.8% over 70 years of age). A sample of 401 participants was required to estimate a 12% incidence of older adults with frailty, with an alpha error of 5% and a statistical power of 95%. In order to control for dropouts, oversampling of 15% was performed, resulting in a final minimum sample of 471 participants. 

In order to use an adequate sampling frame, we decided to recruit participants during the months of highest attendance at primary care centers and contact all the centers of La Ribera Health Department. Meetings were held with the nurses and doctors to explain the study objective, as they are the reference professionals for community-dwelling older adults and could inform potential participants to take part in the study. In addition, posters were made and placed in the waiting rooms of the primary health care centers and community social centers, to inform both the older adults who attended the center and their relatives about the study objectives. Volunteers signed up on a list in each health center, and their data were recorded using alphanumeric codes identifying the center and the individual. Open group discussions were scheduled at the different health centers so that potential participants could ask questions about the study; these information sessions were held in the morning and in the afternoon to facilitate access to all those interested. Finally, all people who showed their willingness to participate and who met the inclusion criteria were included. 

### 2.3. Data Collection

It was necessary to review the participants’ electronic medical records and conduct personalized interviews with each to obtain their comprehensive geriatric assessments along with functional and anthropometric variables. This task was performed by four nurses with at least 10 years of experience in primary health care centers. 

The variables collected from the clinical history were as follows: age; sex; diagnosed comorbidity (hypertension, diabetes mellitus, hyperlipidemia and metabolic syndrome); number of daily active ingredients prescribed; and number of falls recorded in the last 12 months. Other variables were biochemical values within the month preceding the comprehensive geriatric assessment: creatinine (mg/dL), hemoglobin (g/dL), total lymphocytes (×10^3^/mm^3^), total cholesterol (mg/dL), total proteins (mg/dL) and albumin (mg/dL). Participants self-reported their home living situation.

The following five frailty criteria were assessed by nurses in the primary health care centers [5]: weakness (reduced grip strength), slowness (reduced gait speed), weight loss, low physical activity, and exhaustion. Weakness was assessed following standardized procedures and using a KernMap-40 kg dynamometer with a cut-off point grip strength (GS) adjusted for gender and body mass index (BMI). In men, these values were: BMI ≤ 24 kg/m^2^: GS ≤ 29 kg; BMI 24.1–28 kg/m^2^: GS ≤ 30 kg; BMI > 28 kg/m^2^: GS ≤3 2 kg. For women, they were as follows: BMI ≤ 23 kg/m^2^: GS ≤ 17 kg; BMI 23.1–29 kg/m^2^: GS ≤ 18 kg; BMI > 29 kg/m^2^: GS ≤ 21 kg). Slowness was assessed based on the time taken to walk 4.5 m with a cut-off value of < 0.8 m/s. Unintentional weight loss was defined as a loss of 4.5 kg or 5% of body weight in the last year (determined by direct measurement of weight). Low physical activity was defined based on the weighted score of kilocalories expended per week (males: 383 kcal/week and females 270 kcal/week). Lastly, poor endurance and energy were documented from self-reported exhaustion: (a) “I felt that everything I did was an effort”; (b) “I could not get going”. The presence of one or two criteria was considered to indicate prefrailty, and three or more, frailty. The absence of all criteria was considered to indicate robustness. Functional parameters were assessed based on the Barthel index with Cronbach’s alpha 0.70 [27], the Lawton index with Cronbach’s alpha 0.94 [28], and the Tinetti index with Cronbach’s alpha 0.95 [29].

For the nutritional assessment, anthropometric data comprised BMI, hand grip strength determined using a KernMap-40 kg (KERN & SOHN GmbH, Balingen, Germany) dynamometer, body fat percentage determined by bioelectrical impedance analysis (Tanita BC-601, Tanita Europe BV, Amsterdam, The Netherlands), and the MNA-SF (Société des Produits Nestlé S.A, Vevey, Switzerland) with Cronbach’s alpha 0.670 [30]. Abdominal, brachial and thigh perimeters were collected. In addition, we recorded fear of falls (modified Falls Efficacy Scale International, FES-I, α = 0.96) [31]. Health-related QoL was measured using the EQ-5D Index and EQ-5D VAS, according to the parameters of the Spanish population [32]. Lastly, the weekly hours of exercise in the previous 12 months were obtained from verbal reporting by the participants and categorized as: less than 3 h per week, 3 to 6 h per week, and more than 6 h per week.

### 2.4. Ethics

All participants gave their informed consent for inclusion before they participated in the study. The study was conducted in accordance with the Declaration of Helsinki, and the protocol was approved by the Clinical Research Ethics Committee of Hospital Universitario de la Ribera (Valencia, Spain), (Project identification cod HULR_2013/45).

### 2.5. Statistical Analysis

The variables were reported as proportions and/or means and standard deviations (SD). The Kolmogorov-Smirnov test was used to assess normality, and the Levene test was applied to explore homogeneity of variances for continuous variables (age, daily number of drugs prescribed, number of falls in the last 12 months, creatinine, hemoglobin, lymphocytes, cholesterol, protein, albumin, Barthel Index, Lawton Index, Tinetti Index, MMSE, MNA-SF scale, BMI, fat mass, handgrip, abdominal, brachial and thigh perimeters, and the EQ-5D visual analog scale [VAS] and EQ-5D Index). There were no significant outliers. The data met the main assumptions of normality, so the *t*-test for independent samples was used to compare means. The chi-squared test was used to compare categorical variables (gender, comorbidity, home cohabitation, and physical activity).

We analyzed the association between frailty and different risk factors, expressing results as odds ratios (ORs) with their 95% confidence intervals (CIs). Risk factors considered were gender, age > 75 years, living at home alone, arterial hypertension, diabetes mellitus, hyperlipidemia and metabolic syndrome, comorbidity (≥ three diseases), history of falls, polypharmacy (≥ 5 drugs per day), central obesity (waist circumference for women ≥ 88 cm and for men ≥ 102 cm), dependence in the basic activities of daily living (Barthel < 90 points), the instrumental activities of daily living (Lawton ≤ 4) and walking (Tinnetti < 25), obesity (BMI ≥ 30 kg/m^2^), and fear of falling syndrome (FES-I ≥ 20). 

A binary logistic regression model was fitted to explore the importance of the risk factors, defined according to frailty group. Firstly, the complete model, with all the variables in the bivariate analysis, was found to be significantly associated with frailty syndrome. We then used backward stepwise selection to exclude the variables whose elimination from the model failed to produce a significant change (defined as the absence of an adjusted effect of > 10%), including those which did not result in an improved standard error when omitted. In cases where two or more subsets of variables with the same degree of fit were obtained, consensus was sought among the investigators. In order to introduce continuous variables in the regression model, these were categorized according to the cut-offs admitted in the literature. The values were: for hemoglobin, low < 13.5 g/dL in men and < 12.5 g/dL in women, normal ≥ 13.5 g/dL in men and ≥ 12.5 g/dL in women; for total lymphocytes count, low < 1×10^3^/mm^3^, normal 1–4.8×10^3^/mm^3^, high > 4.8×10^3^/mm^3^; for cholesterol, normal < 200 mg/dL, high ≥ 200 mg/dL; for creatinine, low < 0.7 mL/min, normal 0.7–1.3 mL/min, high >1.3 mL/min; for proteins, low < 6 mg/dL, normal 6–8.3 mg/dL, high > 8.3 mg/dL; for albumin, low < 3.4 mg/dL, normal 3.4–5.4 mg/dL, high > 5.4 mg/dL; for central obesity, waist circumference in women ≥ 88 cm and in men ≥ 102 cm; for handgrip following Fried criteria, as above; for BMI, normal < 30 kg/m^2^, high ≥ 30 kg/m^2^; for body fat percentage, high in women ≥ 42% and in men ≥ 30%, normal scores below by gender, and for the MNA-SF, low ≤ 11, normal ≥ 12 points.

## 3. Results

Of the 732 people initially evaluated for eligibility, 22.9% (*n* = 168) were excluded: 95.6% (*n* = 162) declined to participate, and 3.5%, (*n* = 6) did not meet the selection criteria. The final study sample thus comprised 564 participants (77.1%). According to the Fried Frailty Phenotype criteria, the prevalence of frailty was 10.99% (*n* = 62), and the prevalence of prefrailty was 72.89% (*n* = 411), so altogether 83.86% (*n* = 473) of the participants were assigned to the prefrail/frail group (1 to 5 frailty criteria), and 16.13% (*n* = 91) to the robust group (no frailty criteria).

There were more women in the prefrail/frail group. Most participants lived with their partner, although there was a higher percentage of elderly people living at home alone in the prefrail/frail group. The robust group performed a higher level of weekly exercise. There were no differences in the prevalence of comorbidity (Table 1).

Age was higher in the prefrail/frail group (*p* < 0.001). In the functional assessment, the Barthel and Lawton indices showed no differences in the performance of basic and instrumental activities of daily living, respectively, nor were there any differences in walking (assessed by Tinetti) or in cognitive ability (assessed using the MMSE). On the other hand, the prefrail/frail group did present a significantly higher number of falls in the previous 12 months and a significantly higher fear of falling, as assessed by the FES-I (*p* < 0.001). Quality of life was comparably good in both groups (Table 2).

In the subjective nutritional assessment, both groups presented a mean score greater than 11, indicating good nutritional status; however, the prefrail/frail group still had a significantly lower score than the robust group (*p* = 0.019). In the biochemical markers, only hemoglobin differed between groups, with the prefrail/frail group showing lower values (*p* < 0.001). For anthropometric variables, the prefrail/frail group showed a significantly higher BMI and body fat percentage, and a significantly weaker handgrip (Table 3).

**Figure 2:**
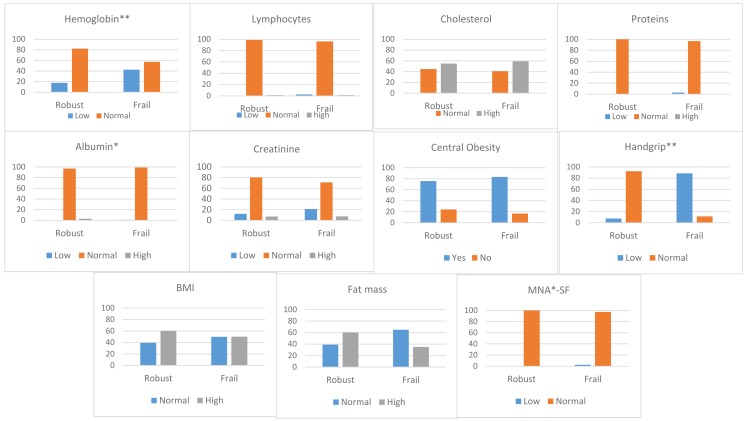
Nutritional assessment in robust vs. prefrail/frail groups, according to established cutoffs for normality. *p < 0.05; **p < 0.001. Cutoff values were: for hemoglobin, low < 13.5 g/dL in men and < 12.5 g/dL in woman, normal ≥ 13.5 g/dL in men and ≥ 12.5 g/dL in women; for total lymphocytes count, low < 1×103/mm^3^, normal 1–4.8×103/mm^3^, high > 4.8×10^3^/mm^3^; for cholesterol, normal < 200 mg/dL, high ≥ 200 mg/dL; for creatinine, low < 0.7 mL/min, normal 0.7–1.3 mL/min, high >1.3 mL/min; for proteins, low < 6 mg/dL, normal 6–8.3 mg/dL, high > 8.3 mg/dL; for albumin, low < 3.4 mg/dL, normal 3.4–5.4 mg/dL, high > 5.4 mg/dL; for central obesity, waist circumference in women ≥ 88 cm and in men ≥ 102 cm; for handgrip following Fried criteria; for BMI, normal < 30 kg/m^2^, high ≥ 30 kg/m^2^; for body fat percentage, high in women ≥ 42% and in men ≥ 30%, normal scores below by gender, and for the MNA-SF, low ≤ 11, normal ≥ 12 points.

For the nutritional assessment, we compared participants based on BMI (Figure 2). Lymphocyte and protein values were mostly in the normal range. The percentage of participants with anemia was higher in the prefrail/frail group (13.73% vs. 42.62% *p* < 0.001). Albumin values were normal in most of the sample. In both groups, there was a high percentage with hypercholesterolemia (robust 55.1% vs. frail 59.2%), central obesity (robust 75.8% vs. frail 83.3%) and obesity (robust 60.4% vs. frail 50.1%). However, the percentage of participants with high body fat was lower in the prefrail/frail group (60.4% vs. 35%), and the percentage with low palm grip strength was higher (7.7% vs. 88.6%). Nutritional status, as assessed by the MNA-SF, was adequate in 2.7% of the frail elderly.

Comparing risk factors between groups, the prefrail/frail participants showed higher odds of presenting, in order of magnitude: a fear of falling, a recent history of falls, anemia (hemoglobin < 13.5 g/dL in men and < 12.5 g/dL in women), age over 75, living at home alone, polypharmacy, and female gender (Table 4).

A binary logistic regression was performed to identify predictors of frailty. The following variables were entered into the model: age group, female gender, living at home alone, diabetes mellitus, polypharmacy, Barthel < 90, previous falls, FES-I ≥ 20, anemia (hemoglobin < 13.5 g/dL in men and < 12.5 g/dL in women), central obesity, obesity, and physical activity. Handgrip was not introduced because it is a criterion of the frailty phenotype. The model was statistically significant (*p* < 0.001; *R*^2^ = 0.43), correctly classifying 83.9% of cases, with a sensitivity of 94.4% and a specificity of 39.2%.

The variables included in the final model were female gender, age over 75 years, anemia, history of falling, and fear of falling. The strongest predictor of frailty was fear of falling. Physical activity was a determining factor for preventing frailty syndrome (Table 4).

## 4. Discussion

Our results indicate that frailty in functionally independent older people is associated with being a woman, age of 75 years or older, anemia, a recent history of falling, fear of falling, and lack of physical activity. As a geriatric syndrome, frailty is associated with increasing age, but malnutrition and decreased functionality are also related. Erroneously, many clinicians consider any adult over 80 years of age who presents dependence, malnutrition, and comorbidity to be frail, but these may be risk factors or consequences [7]. Most studies that analyze malnutrition and its relationship with frailty are conducted in older people in institutions or hospitals, but functionally independent community elders are also at risk for frailty and other geriatric syndromes [8,9]. The aim of this study was to determine the factors associated with frailty in community-dwelling older people with functional independence and no cognitive impairment, and to analyze the relationship with nutritional status. 

Our sample had a lower prevalence of frailty and a higher prevalence of prefrailty than in similar studies [24,33,34,35]. However, there are clear difficulties when comparing epidemiological figures on frailty, stemming from the different concepts and categorizations used, such as the phenotype of frailty and the subtypes of frailty, as well as the population characteristics analyzed [6,24,34,36]. The sociodemographic and functional characteristics in our population were similar to those for other studies in older adults living in rural communities [35], that is, older people with functional independence in basic and instrumental activities of daily life, predominantly living at home with their partner, presenting low comorbidity and consequently high perception of health-related quality of life [33]. The loss of functionality is more accentuated in the frail population [5,12], so the selection of a sample with functional independence could condition the recruitment of a greater number of prefrail older adults. Clinical assessment of frailty and its prevention should start from the prefrail stages, since these people are at the greatest risk of increasing their frailty status and functional loss [12]. However, clinicians may underestimate this risk in daily clinical practice, instead erroneously considering patients with more advanced age and greater dependence and comorbidity to be frail [7]. 

Malnutrition is related to frailty [22,37]. For this reason, international clinical practice guidelines [20,21] advise a broad nutritional assessment as part of the appropriate approach to frailty, although there are still gaps in understanding how to implement management strategies for different populations [22]. 

Our sample presented a status of overnutrition according to different nutritional markers, including obesity according to BMI, waist circumference, body fat percentage, and biochemical markers. While BMI alone does not seem to be a suitable measure for assessing the risk of frailty [38], in populations with high caloric intake there is evidence of an association between obesity and frailty [39], and BMI trajectories have revealed that both long-term obesity and onset of obesity in late adulthood were associated with frailty [40]. Central obesity, as assessed by waist circumference, was high in both groups, although the body fat percentage was slightly lower in the prefrail/frail group. These parameters likewise do not seem to be suitable for predicting frailty [38], although several studies have found a relationship between high waist circumferences and frailty [18]. Comparisons between some nutritional parameters are problematic due to the diversity of globally standardized measures and physiological changes during the aging process [19], but the only anthropometric parameter showing alterations in the prefrail/frail group was handgrip. As handgrip strength is a criterion of the phenotype of frailty [5], though, it is logical that a larger proportion of participants in the prefrail/frail group presented loss of handgrip strength.

Both groups showed a good nutritional status (above 11 points) on the MNA-SF, although in the prefrail/frail elderly the figures were lower than in the robust group. Recent studies indicate that the MNA-SF, a tool for screening for malnutrition or risk of malnutrition [30], is a good predictor of frailty [38,41]. That said, it is not capable of detecting overnutrition, so for the analyzed sample, it may not be adequate. The right screening tools for the population under study is essential, as is the performance of screening in all the dimensions previously indicated (analytical, anthropometric, and subjective reporting) since there is no single nutritional parameter that capable of accurately classifying nutritional status by itself [23].

Biochemical markers also indicated good nutritional status in terms of lymphocytes, total protein, albumin, and creatinine, but there was also a large proportion of cholesterolemia in both groups. In contrast, the prevalence of anemia was higher in the prefrail/frail group. There is evidence that deficits in the daily amount of ingested energy or in macro and micronutrients like total proteins, albumin, creatinine, essential amino acids, vitamin D or hemoglobin, could be predictors of frailty [14], but more studies are still necessary in certain populations, such as the one analyzed, since functional independence in basic and instrumental activities, despite frailty, would allow older people to shop and cook according to their tastes [8], thus maintaining optimal nutritional status.

After including the risk factors in the regression model, female sex and age over 75 years were both predictors for frailty, in consonance with the literature [42]. Anemia also turned out to have a high predictive capacity, suggesting that screening for this biomarker and implementing interventions to reverse it, for example by increasing exercise, could contribute to preventing frailty, since for every 1 g/dL increase in hemoglobin, the risk of frailty decreases by 4% [43,44]. Falls and fear of falling were also included in the model, with fear of falling being the most predictive. Falls, too, are directly related to frailty [11], with most studies indicating that falls are a consequence of frailty and prefrailty [45,46]. On the other hand, there is no clear relationship between fear of falls and risk of frailty [47]. Rather, this fear is associated with older people curtailing some activities due to a lack of confidence. This limits the performance of advanced activities of daily life, such as dancing or exercise, which in turn accelerates the loss of functional, cognitive and social dimensions [48]. Physical activity clearly helps prevent frailty as well as loss of function and cognition, and it is favorable for people’s emotional state and quality of life. In contrast, sedentarism is not only a predictor of frailty, but also of functional loss, cardiovascular risk, and mortality [49,50].

Living alone at home, despite being an independent factor related to frailty, was not included in the final predictive model. This can be explained by the likelihood that cooking for oneself, eating alone, and losing the chance to use meals as a source of play and family communication decreases people’s interest in buying and cooking certain foods, which is in itself a risk factor [51]. Similarly, comorbidity and polypharmacy were also not predictors of frailty despite their clear relationship in the literature. This may be because the types of comorbidity in our sample were associated with less impairment of functionality, which is also reflected in the high perception of quality of life reported by the sample in both groups [33]. Likewise, the other nutritional parameters did not prove to be predictors of frailty. The literature indicates that frailty is related to malnutrition, both excess and deficit [14], although most studies focus on the stronger relationship with malnutrition [5,16,52] rather than overweight and obesity.

This study has a number of limitations, the first being that it is a cross-sectional study. It was not possible to include data on daily caloric intake nor to analyze the macro or micronutrients ingested. We were similarly unable to analyze all the parameters recommended in the nutritional assessment due to the difficulty of obtaining them from the community population. Although the population analyzed is considered to have a middle to high income, we did not specifically analyze socioeconomic level. In addition, there is a difference in sample size between the groups analyzed according to different prevalence of frail phenotype in the sample and this, coupled to the study’s inclusion criteria, could difficult the generalizability of the findings. Finally, it was not possible to analyze the type of physical exercise the participants engaged in, as the research team did not witness these activities.

The studies with the highest prevalence of malnutrition are carried out in hospitalized and institutionalized older people [13,14]. There is a pending need for longitudinal studies that include functionally independent, community-dwelling older people in order to assess their nutritional status and better understand the role of malnutrition and overnutrition in the risk of frailty.

## 5. Conclusions

Functionally independent community-dwelling older people are at risk for frailty, falls, and other geriatric syndromes. The prevalence of frailty and prefrailty in community populations with excess malnutrition was 10.99% and 72.89%, respectively. Female sex, advanced age, anemia, a recent history of falling, fear of falling, and level of sedentary or light physical activity should be considered risk factors for frailty in functionally independent older people.

## Figures and Tables

**Table 1:** Participant characteristics: sociodemographics, cohabitation, morbidity, and physical activity.

		Robust Group (*n* = 91)	Prefrail/Frail Group (*n* = 473)	*p*-Value *
Gender	Women	31 (34.1)	325 (68.7)	<0.001
	Men	60 (65.9)	148 (31.3)	
Cohabitation	Partner	70 (76.9)	288 (60.9)	<0.001
	Alone	15 (16.5)	141 (29.8)	
	Children or other relatives	6 (6.6)	44 (9.3)	
Physical activity	<3 h/week	0 (0)	228 (48.2)	<0.001
	3–6 h/week	41 (45.1)	141 (29.8)	
	>6 h/week	50 (54.9)	104 (22)	
Comorbidity	Hypertension	60 (65.9)	310 (65.5)	0.942
	Diabetes mellitus	20 (22)	142 (30)	0.120
	Hyperlipidemia	36 (36.9)	207 (43.8)	0.458
	Metabolic syndrome	14 (15.4)	89 (18.8)	0.438

* *p*-value: obtained from Chi-square test.

**Table 2:** Age, functional, and cognitive characteristics in robust versus prefrail/frail participants.

Variable	Robust *N* = 91	Prefrail/Frail *N* = 473			
	Mean (SD)	Mean (SD)	Mean Difference	95% CI	*p*-Value *
Age, years	74.37 (3.23)	76.37 (4.02)	−1.99	−2.76, −1.23	<0.001
Daily prescribed drugs, *n*	3.91 (2.61)	4.86 (3.09)	−0.95	−1.63, −0.27	0.006
Barthel, points ^1^	94.89 (7.96)	93.45 (9.87)	1.44	−0.71, 3.60	0.189
Lawton, points ^2^	7.46 (0.95)	7.30 (1.17)	0.17	−0.06, 0.39	0.145
Tinetti, points ^3^	25.96 (3.23)	25.75 (3.36)	0.20	−0.55, 0.95	0.595
MMSE, points ^4^	27.45 (1.50)	27.87 (1.69)	−0.42	−1.14, 0.31	0.261
Falls over previous 12 months, *n*	0.23 (0.67)	0.79 (1.41)	−0.56	−0.75, −0.37	<0.001
FES-I, points ^5^	10.90 (11.78)	20.27 (17.07)	−9.37	−12.26, −6.48	<0.001
EQ-5D Index, points ^6^	0.80 (0.22)	0.79 (0.22)	0.01	−0.43, 0.06	0.787
EQ-5D VAS, points ^7^	75.82 (20.29)	74.94 (20.77)	0.89	−3.77, 5.55	0.708

CI: confidence interval; SD: standard deviation. ^1^ The Barthel Index assesses function referred to basic activities of daily living based on a score of 0 to 100. ^2^ The Lawton Index assesses function referred to instrumental activities of daily living based on a score of 0 to 8. ^3^ The Tinetti Scale evaluates gait and balance based on a score of 0 to 28. ^4^ The Mini Mental State Examination (MMSE) assesses cognitive function based on a score of 0 to 30. ^5^ The Falls Efficacy Scale International assesses fear of falling on a score of 0 to 28. ^6^ EuroQoL 5 dimensions (EQ-5D) Index assesses objective health quality of life on a score of 0 to 1 ^7^ EuroQoL 5 dimensions (EQ-5D) visual analog scale (VAS) assesses subjective health quality of life on a score of 0 to 100. * *p*-value: obtained by *t*-test.

**Table 3:** Differences between groups in nutritional status.

Variable	Robust Group		Prefrail/Frail Group		Mean Difference	95% CI	*p*-Value *
	*N*	Mean (SD)	*n*	Mean (SD)			
MNA-SF, points ^1^	91	13.69 (0.49)	473	13.55 (0.73)	0.14	0.02, 0.27	0.019
Hemoglobin, g/dL	79	14.69 (1.38)	413	13.63 (1.42)	1.06	0.71, 1.40	<0.001
Total lymphocyte count, ×10^3^/mm^3^	79	2.26 (0.84)	407	2.32(2.43)	−0.05	−0.60, 0.49	0.850
Total cholesterol, mg/dL	78	194.95 (35.21)	400	192.15 (36.94)	2.80	−6.12, 11.72	0.537
Total proteins, mg/dL	64	7.06 (0.34)	345	7.32 (5.05)	−0.25	−1.50, 0.99	0.690
Albumin, mg/dL	34	4.56 (0.45)	196	4.37 (0.83)	0.18	−0.11, 0.47	0.220
Creatinine, mg/dL	82	1.00 (0.25)	423	0.95 (0.38)	0.05	−0.04, 0.13	0.261
BMI, kg/m^2^	91	29.08 (3.45)	473	30.25 (4.61)	−1.16	−1.99, −0.34	0.006
Waist circumference, cm	91	103.76 (9.37)	473	104.18 (11.66)	−0.42	−2.63, 1.79	0.709
Brachial circumference, cm	91	30.12 (3.15)	473	30.60 (3.78)	−0.48	−1.22, 0.25	0.197
Thigh circumference, cm	91	46.63 (4.93)	473	47.23 (6.68)	−0.60	−2.05, 0.84	0.411
Handgrip strength, kg	91	31.17 (6.85)	473	18.29 (7.57)	12.88	11.20, 14.55	<0.001
Body fat %	91	34.64 (6.49)	471	39.40 (7.07)	−4.77	−6.34, −3.20	<0.001

BMI: Body Mass Index; CI: confidence interval; SD: standard deviation. ^1^ The MNA-SF Mini Nutritional Assessment Short Form evaluates undernutrition based on a score of 0–14. * *p*-value: obtained by *t*-test.

**Table 4:** Odds ratios and 95% confidence intervals from bivariate analysis and multivariate logistic regression analysis of risk factors related to frailty.

Factor	Crude OR (95% CI)	*p* Value *	Adjusted OR (95% CI)	*p* Value **
Female gender	1.25 (2.64, 6.83)	<0.001	2.37 (1.24, 4.53)	0.009
Age > 75	2.33 (1.46, 3.71)	<0.001	3.31 (1.76, 6.21)	<0.001
Living at home alone	2.15(1.19, 3.87)	0.007		ns
Hypertension	0.98 (0.61, 1.57)	ns		
Diabetes mellitus	1.52 (0.89, 2.59)	ns		
Hyperlipidemia	1.19 (0.75, 1.88)	ns		
Metabolic syndrome	1.27 (0.69, 2.35)	ns		
≥3 comorbidities	1.27 (0.69, 2.35)	ns		
Polypharmacy (≥ 5 drugs per day)	1.65 (1.03, 2.62)	0.032		ns
Activities of Daily Living, Barthel <90 points	1.71 (0.23, 3.56)	ns		
Instrumental. Activities of Daily Living Lawton ≤ 4 points	0.96 (0.11, 8.33)	ns		
Gait and speed, Tinetti < 25 points	1.24 (0.77, 2.08)	ns		
Previous fall in the last 12 months	3.89 (2.1, 7.19)	<0.001	1.91 (1.13, 3.25)	0.016
Fear of falling, FES-I ≥ 20 points	4.88 (2.53, 9.14)	<0.001	4.01 (1.76, 9.16)	0.001
Hemoglobin, low	3.45 (1.87, 6.34)	<0.001	2.45 (1.19, 5.03)	0.015
Central obesity	1.59 (0.93, 2.72)	ns		ns
Obesity (BMI ≥ 30 kg/m^2^)	1.52 (0.96, 2.40)	ns		ns
Body fat percentage, high	1.21 (0.76, 1.92)	ns		
Physical activity (≥3 h/week)	0.31 (0.12, 0.38)	<0.001	0.23 (0.15, 0.35)	<0.001

BMI: body mass index; CI: confidence interval; OR: odds ratio. FES-I Falls Efficacy Scale International; hemoglobin low < 13.5 g/dL in men and < 12.5 g/dL in women; central obesity: women waist circumference ≥ 88 cm and men ≥ 102 cm; high body fat percentage: women ≥ 42% and men ≥ 30%; ns: non statistic significatively. * *p*-value: OR was obtained by binary regression model between frailty and other study variables. ** *p*-value: adjusted OR was obtained by multivariable regression model between frailty and statistic significance variables.
